# Supplementary material for: Bcl-2 Up-Regulation Mediates Taxane Resistance Downstream of APC Loss
Source: Int J Mol Sci. 2024 Jun 19;25(12):6745. doi: 10.3390/ijms25126745 (PMC11203545; doi:10.3390/ijms25126745)
Supplement: Supplementary file 1 [file ijms-25-06745-s001.zip › ijms-3050710-supplementary.pdf]

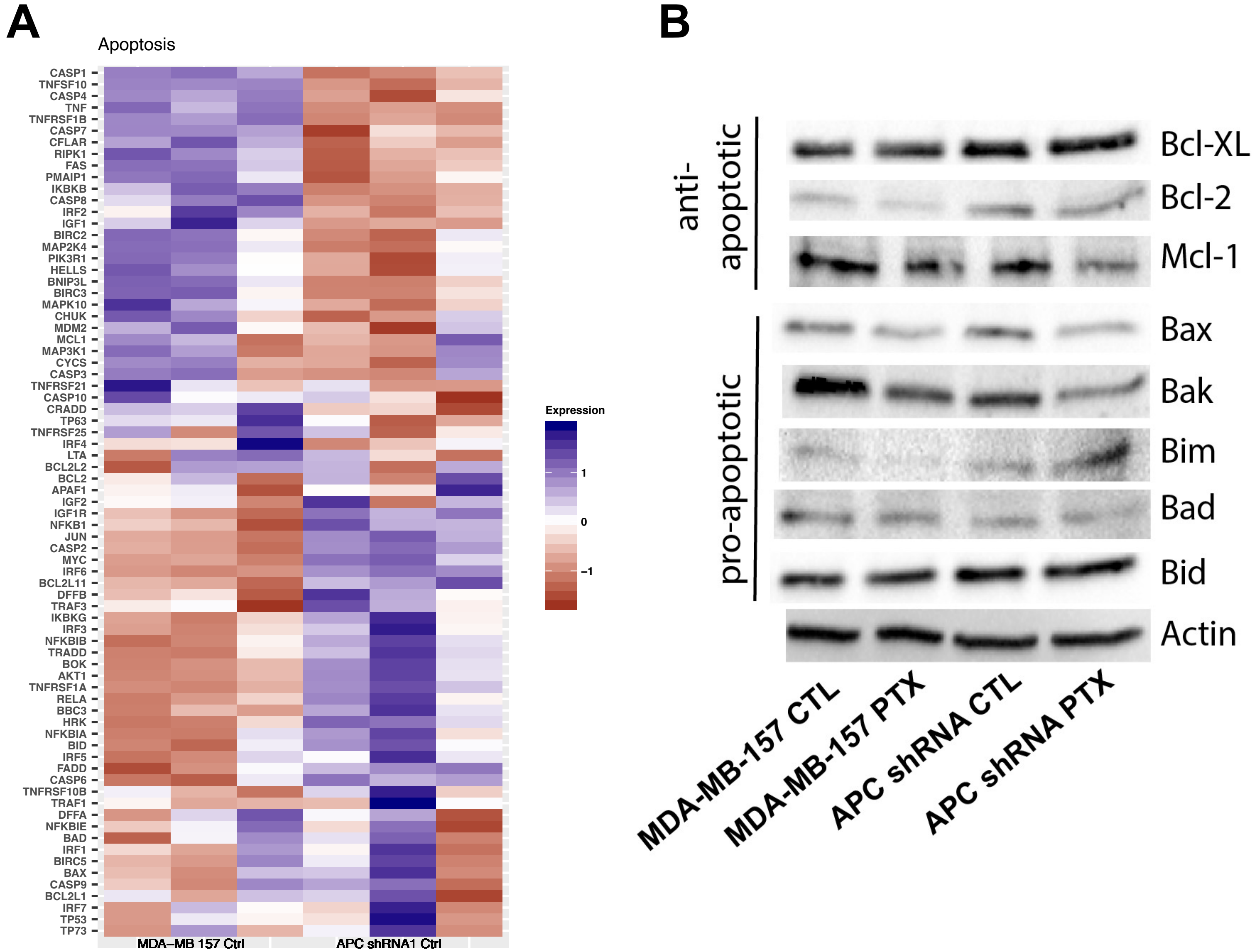

Supplementary Figure S1. A) Heat-map of transcripts involved in apoptosis in the MDA-MB-157 model with APC knockdown. B) Pro- and anti-apoptotic members of the BCL-2 family were assessed by western blot in cells control or PTX treated for 24 hrs. Experiments were run three times and representative blots are shown.
